# Transcutaneous Electrical Stimulation of Acupoints Changes Body Composition and Heart Rate Variability in Postmenopausal Women with Obesity

**DOI:** 10.1093/ecam/nep145

**Published:** 2011-02-20

**Authors:** Li-Wei Chien, Miao-Hsiang Lin, Hsueh-Yu Chung, Chi-Feng Liu

**Affiliations:** ^1^Department of Obstetrics and Gynecology, Taipei Medical University and Hospital, Tàipei, Taiwan; ^2^Graduate student of Department of Nursing, National Taipei College of Nursing, Taipei, Taiwan; ^3^Jen-Teh Junior College of Medicine, Nursing, and Management, Miaoli, Tàipei, Taiwan; ^4^Graduate Institute of Integration of Traditional Chinese Medicine with Western Nursing, National Taipei College of Nursing, Taipei 11211, Taiwan

## Abstract

This study aimed to evaluate the effect of transcutaneous electric acupoint stimulations (TEAS) on body composition and heart rate variability (HRV) in postmenopausal women with obesity. In this prospective study, 49 postmenopausal women were recruited in Taiwan. Body composition was used as a screening test for obesity (percentage body fat > 30%, waist circumference > 80 cm). The experimental group (*n* = 24) received TEAS treatment 30 min twice per week for 12 weeks at the Zusanli (ST 36) and Sanyinjiao (SP 6) acupoints. The control group (*n* = 25) did not receive any intervention. The study of HRV was analyzed by time (standard deviation of the normal-to-normal (NN) intervals (SDNN) and square root of the mean squared differences of successive NN intervals (RMSSD) indices) and frequency domain methods. Power spectral components were obtained at low (LF) and high (HF) frequencies. Body composition and HRV values were measured at the 4th, 8th, and 12th weeks. A total of 40 subjects completed this study. Waist circumference and percentage body fat in the experimental group (*n* = 20) were significantly less than those of the control group (*n* = 20) at the 8th and 12th weeks (all *P* < .05). Additionally, at the same time points, percentage lean body mass in the experimental group was significantly greater than that in the control group (*P* < .05). SDNN values increased significantly at the 4th and 8th weeks when compared with the control group (all *P* < .05). At 12 weeks, SDNN value was not significantly different from that of the control group (*P* = .105). TEAS treatment improves body composition, and has a transient effect on the HRV in postmenopausal women with obesity.

## 1. Introduction

In 2005, *∼*1.6 billion adults worldwide were overweight, and at least 400 million adults were obese. World Health Organization projections indicate that by 2015, *∼*2.3 billion adults will be overweight and more than 700 million will be obese [1]. In Taiwan, the prevalence of obesity (BMI ≥ 25) in men is 24.8% and in women 25.5% [2].

The prevalence of being overweight generally arises between the ages of 20 and 60 years, and overall it seems that while men gain more body weight earlier in life, women tend to accumulate more body weight during the transition through menopause [3–5]. The increase in adiposity during menopause seems to be a consequence of the decline in endogenous estrogens and reduced energy expenditure [4, 6]. Redistribution of body fat with increased visceral fat is believed to correlate with increased prevalence of metabolic syndrome, and consequently, cardiovascular disease in postmenopausal women [7, 8].

The autonomic nervous system plays an important role in the regulation of energy expenditure and body fat storage [9, 10]. Studies have shown that regional body fat distribution varied with the heart rate variability (HRV) of obese subjects and weight loss increased parasympathetic control of HRV [11, 12]. Reduced sympatho-vagal activity has also been observed in women after menopause [13], and is associated with higher body fat content, blood pressure and blood lipid concentrations in postmenopausal women [14]. Accordingly, modulation of sympathetic and/or parasympathetic activity may play a role in regulating body fat composition, lipid metabolism, and cardiovascular profiles after menopause.

Acupuncture has been applied extensively around the world as an alternative treatment to reduce body weight [15]. The underlying mechanisms of acupuncture in weight reduction might result from reducing appetite [16] and mobilizing the body energy depots through lipolytic effect [17]. Several studies have suggested that acupuncture and acupuncture point procedures could also modulate the activities of the sympathetic and parasympathetic nerves [18, 19], with an effect of increased vagal modulation [20, 21]. Site-specific effects of acupuncture on gastric motility have been demonstrated in rats, and HRV analysis is useful to evaluate the efficacy of acupuncture on the activity of autonomic nervous system [22].

The purpose of this study was to evaluate the effect of transcutaneous electrical acupoint stimulation (TEAS) on body composition and HRV in postmenopausal women with obesity. The Zusanli (ST 36) and Sanyinjiao (SP 6) were selected as main acupoints in the treatment group.

## 2. Methods

### 2.1. Subjects

This was a prospective study conducted from November 2006 to June 2007. All study procedures were approved by the committee review board at the National Taipei College of Nursing. A total of 49 female volunteers were recruited from communities in Taipei. Written consent was obtained before their participation in the study. Initial evaluation included measurements of body composition (i.e., body weight, waist circumference, hip circumference, waist-hip ratio, percentage body fat and percentage lean body mass). Weight was measured to the nearest 0.1 kg using an electronic scale. Waist circumference was measured at the level midway between the lateral lower rib margin and iliac crest. Hip circumference was measured at the levels of the trochanters in the standing position. A bioelectrical impedance analyzer system (BT-905 Body Composition Analyzer, Skylark Co. Ltd., Taiwan) was used to measure body fat and lean mass. All participants met the following criteria: (i) had experienced menopause naturally at least 1 year previously, (ii) <64 years of age, (iii) >30% body fat, and (iv) waist circumference > 80 cm. Individuals with the following conditions were excluded: (i) gynecological disease including hysterectomy and/or oophorectomy, (ii) history of cancer, (iii) cardiac arrhythmia or an implanted cardiac pacemaker, (iv) paralysis of lower limbs or decreased sensation, and (v) inflammation of skin defects exhibited in skin of lower limbs.

### 2.2. Groups and Intervention

The 49 women were randomized into the treatment (*n* = 24) and control groups (*n* = 25). A TEAS device using interferential current (Skylark 730IF, Skylark Device & Systems Co. Ltd, Taiwan) was applied. Its specifications are as follows: maximum output current 50 mA, output voltage 0–25 V and wave width 125 *μ*A; two different frequency modulations (FM) are mixed, the frequency of channel one set at 4000 Hz and channel two at 61 Hz. The treatment was 30 min per session, two sessions per week for 12 weeks using two surface electrodes, one to the Zusanli (ST36) and the other to Sanyinjiao (SP6) acupoint. We used close-by point to complete an electric circuit rather than use the point in the opposite extremity, thereby letting the current pass through the cardiac region. The control group did not receive any intervention. Measurements of body composition and HRV that were performed at the initial evaluation were repeated at the 4th, 8th, and 12th week.

### 2.3. HRV Analysis

HRV was recorded in the sitting position after a 5-min rest and in a quiet room at room temperature using the Heart Rate Variability Analyzer (SA-3000P, Medicore Co. Ltd, Seoul, Korea). The sensor of HRV was clipped on the index finger (2nd finger) of the left hand. The subjects were asked to keep silence and stay inactive during the measurement. The subjects were earlier told to avoid strenuous physical activity, and alcohol and coffee intake within 24 h before the measurement. HRV analysis was performed according to the guidelines of the Task Force of the European Society of Cardiology [23]. The following time domain parameters were evaluated: SDNN (ms), that is, the standard deviation of the normal-to-normal (NN) intervals; RMSSD (ms), that is, the square root of the mean squared differences of successive NN intervals. SDNN reflected overall HRV, whereas RMSSD was considered to be an index of parasympathetic modulations of heart rate. The frequency domains of HRV components were obtained at low (LF 0.04–0.15 Hz) and high (HF 0.15–0.4 Hz) frequencies, in absolute units (ms^2^), and the normalized units were computed by dividing the absolute power of a given LF or HF component (ms^2^) by the total power minus very low frequency (0.003–0.04 Hz) power and then multiplying this ratio by 100. The LF domain reflects both the sympathetic and the parasympathetic nervous systems and the HF domain primarily reflects vagal cardiac control; the LF/HF ratio was calculated to determine the sympatho-vagal balance [23].

### 2.4. Data Analysis

Power analysis by SSIZE software was used to calculate the sample size, with significance level at 0.05; the power of the test of 0.8 showed that 22 women were needed in each group. Continuous variables were expressed as median and range. Age and duration of menopause were also expressed as median and range because they did not follow a normal distribution. For the categorical variables, the frequency and the percentage were shown. The Mann-Whitney *U*-test for continuous variables and the chi-square test for categorical variables were used to test for differences between the two groups at baseline. A mixed model was established to examine the effect on a group at each time point and the time trend for a given group. Bonfferoni correction with an adjusted *α* level of 0.0167 (*α*′ = 0.05/3 = 0.0167) was used to test the difference between a time point and the baseline. All analyses were performed by SAS version 9.1.3 software (SAS Inc, Cary, NC). *P* < .05 was defined as the significance level.

## 3. Results

### 3.1. Demographic and Anthropometric Parameters

**Figure 1:**
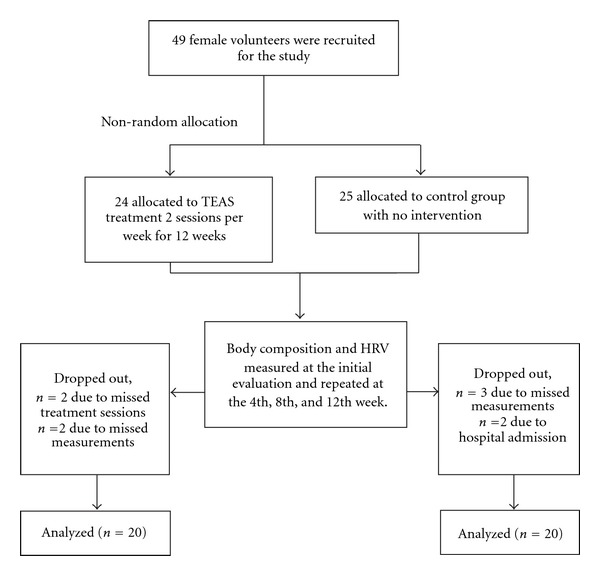
Flow chart of the distribution of the cohort study.

A total of 40 subjects completed this study (Figure 1). Four subjects in the experimental group and five subjects in the control group dropped out due to missed treatment sessions (*n* = 2), missed measurements (*n* = 5), and hospital admission (*n* = 2). Baseline characteristics are summarized in Table 1. At baseline, the epidemiological characteristics of both groups were similar (*P* > .05), but the median age of the experimental group was greater than that of the control group (*P* = .004). The median age and the median duration of menopause of the control group were 52.5 and 2.5 yrs, respectively. The median age and the median duration of menopause of the experimental group were 57 and 5 yrs, respectively. 

The results of mixed model analysis with covariates for body composition are shown in Table 2. The body compositions of the two groups were similar at baseline (*P* > .05). After adjustment for age, occupation, and working stress, the mixed model analysis indicated significant differences in waist circumference, percentage body fat, and percentage lean body mass between the control and experimental groups at the 8th and the 12th week (*P* < .05). At the 8th week, the waist circumference and the percentage body fat of the experimental group were less than those of the control group (*P* = .028 and .002, resp.). At the 12th week, when compared with the control group, the waist circumference and the percentage body fat of the experimental group were less (*P* = .006 and <.001, resp.) and the percentage lean body mass was greater (*P* < .001).

### 3.2. HRV

Table 3 presents the analytical results for HRV parameters by the mixed model. When compared with the control group, the SDNN and the total power were higher at the 4th week (*P* = .024 and .049, resp.). The SDNN was also greater for the experimental group (*P* = .029) at the 8th week. However, the SDNN for the experimental group was not elevated significantly at the 12th week (*P* = .105). The changes in the LF (nu), HF (nu) and LF/HF ratio from baseline to 12th week were not statistically different between the two groups.

### 3.3. Discussion

Our study showed that at the 8th and 12th weeks of TEAS treatment, waist circumference and percentage body fat in the experimental group were significantly less than those of the control group. The treatment did not result in weight loss or a smaller hip circumference than the non-treatment group. This might be explained by the fact that women in the treatment group demonstrated an increase in lean mass. Our data are consistent in that waist circumference was the most affected variable in the previous trials of electro-acupuncture treatment in obese women [24, 25]. Previous studies of acupuncture for the treatment of obesity have focused on weight reduction and most trials showed only modest effect on the weight loss [15]. The direct effect of acupuncture on body fat composition has not been explored before. We found that TEAS was able to reduce fat mass, increase lean body mass and reduce waist circumference.

We also demonstrated that a decrease of body fat mass and waist circumference was accompanied with a withdrawal of the sympathetic activity indicated by the increase of SDNN: this parameter is negatively influenced by the sympathetic compound of the autonomic nervous system [23]. The increase in the component of HRV at 4 and 8 weeks was probably not due to the effect of weight loss on the sympathetic activity because there were no significant differences in body weight between the treatment and control groups. As a result, these changes may indicate a shift of sympathetic-vagal balance toward reduced sympathetic tone and vagal predominance after TEAS treatment. At the 12th week of treatment, we observed a partial sympathetic reactivation, as indicated by no significant changes of SDNN as compared with the control groups.

It is well established that the coordination of energy homeostasis relies on the normal functioning of the sympathoadrenal system [9, 10]. Our study confirms the results of a previous study that showed reduced sympathovagal activity to be associated with higher postmenopausal body fat content [13]. The dysregulation of energy metabolism could induce an increase in total adiposity and a redistribution of fat to the abdominal region [3–5]. In obese women, reduction of body weight can increase parasympathetic control of HRV significantly [13], but the increase is temporary if body weight does not remain controlled [26]. We found that waist circumference and percentage body fat of the experimental group remained less than those of the control group at the 12th week, despite the restoration of cardiac autonomic function. This may suggest a local effect of TEAS on the abdominal fat in favor of adipose tissue lipolysis.

The treatment principles of Traditional Chinese Medicine for obesity are reinforcing Qi, resolving dampness, invigorating the spleen, and nourishing the kidney [27]. The primary acupoints used in this study were Zusanli (ST36) and Sanyinjiao (SP6). The Zusanli was used for improving the stomach and invigorating the spleen to remove dampness and phlegm. The Sanyinjiao was used for eliminating water and dampness. Stimulation of acupoints improves Qi and blood circulation [28] and subsequently activates the metabolism to increase energy consumption [29].

Both auricular and somatic acupoints have been shown to be effective in the acupuncture treatment of obesity [15]. The modes of stimulation include manual needling, mechanical pressure (acupressure), heating (moxibustion) and transcutaneous electrical nerve stimulation (TENS). Electrical stimulation applied on needles inserted into the acupoints has the advantage of using precisely identified frequency and intensity during the acupuncture treatment [30]. Evidence from the rat experiment indicated that electroacupuncture at body points produced a significant reduction of both food intake and the body weight, with 2 Hz more effective than 100 Hz. Electroacupuncture could also produce a reduction of plasma level of total cholesterol and triglyceride, with 100 Hz more effective than 2 Hz. It is supposed that the 2/100 Hz alternative mode of stimulation may have dural effect on both appetite and fat metabolism, but this still lacks convincing clinical evidence [31]. Medium-frequency alternating currents defined as currents in the frequency range from 1 to 10 kHz, are used mainly in rehabilitation [32]. Transcutaneous stimulation using medium-frequency alternating currents provides a higher proportion of electrical energy to stimulate the underlying tissue than the conventional TENS device [33]. However, further studies in comparing their effectiveness are warranted. Using a portable device of TENS as used in this study may provide an alternative for individuals who want to manage the weight loss for a longer period of time.

The limitations of this study include the small sample size, relatively short study period, and the lack of adequate control for possible placebo effects. Also, a subject's mental status, setting, room temperature, body position, mood and movement all influence heart rate. Setting and room temperature were controlled, but it was difficult to account for a patient's mood or mental status. Since the study was cross-sectional, we cannot make causal inferences about relationships between obesity and postmenopausal status. And lastly, treating fixed acupoints as this study might not fully demonstrate the effect of TEAS. More acupuncture points should be considered in further investigations.

## 4. Conclusions

Transcutaneous electrical stimulation of the Zusanli (ST 36) and Sanyinjiao (SP 6) acupoints was effective in reducing percentage body fat and waist circumference in postmenopausal women during a period of 12 weeks. This effect may be related to modulation of the autonomic nervous system and reflected in increased HRV components. Further study is warranted, especially, in the evaluation of long-term effect.

## Figures and Tables

**Table 1:** Characteristics of study subjects.

Variables	Control (*n* = 20)	Experimental (*n* = 20)	*P*-value
Age (years)^a^	52.5 (48.0, 61.0)	57.0 (49.0, 63.0)	.004*
Duration of menopause (years)^a^	2.5 (1.0, 10.0)	5.0 (1.0, 10.0)	.148
Marriage^b^			1.000
Married	20 (100.00)	19 (95.00)	
Widow	0	1 (5.00)	
Occupation^b^			.004*
No	19 (95.00)	11 (55.00)	
Yes	1 (5.00)	9 (45.00)	
Exercise^b^			.457
Never	4 (20.00)	1 (5.00)	
Occasional	7 (35.00)	8 (40.00)	
Regular	9 (45.00)	11 (55.00)	
Tea^b^			.693
No	3 (15.00)	4 (20.00)	
Occasional	5 (25.00)	7 (35.00)	
Frequently	12 (60.00)	9 (45.00)	
Coffee^b^			.236
No	9 (45.00)	7 (35.00)	
Occasional	3 (15.00)	8 (40.00)	
Frequently	8 (40.00)	5 (25.00)	
Medical history^b^			.487
No	9 (45.00)	5 (25.00)	
Yes	11 (55.00)	15 (75.00)	
Medications^b^			1.000
No	14 (70.00)	14 (70.00)	
Yes	6 (30.00)	6 (30.00)	
Health food^b^			.465
No	6 (30.00)	4 (20.00)	
Yes	14 (70.00)	16 (80.00)	
Stress^b^			.046*
Light	14 (70.00)	6 (30.00)	
Medium	5 (25.00)	9 (45.00)	
Heavy	1 (5.00)	5 (25.00)	

Data are presented as median (range) or number (%).

^a^Mann-Whitney *U*-test was used; ^b^Chi-square test was used.

*Significantly different, *P* < .05.

**Table 2:** Body composition of the two study groups by mixed model.

Variables	Control (*n* = 20)	Experimental (*n* = 20)	*P-*value
Body weight (kg)			
Baseline	64.52 ± 5.97	63.48 ± 4.90	.752
4th week	65.05 ± 6.11	62.93 ± 4.78	.495
8th week	65.46 ± 6.10	62.67 ± 4.66	.219
12th week	65.68 ± 6.01	62.40 ± 4.81	.192
Waist circumference (cm)			
Baseline	84.25 ± 3.55	84.25 ± 2.69	.977
4th week	84.93 ± 4.21	83.50 ± 2.61	.525
8th week	85.60 ± 3.76	82.80 ± 2.56	.028*
12th week	85.78 ± 3.56	82.33 ± 2.75	.006*
Hip circumference (cm)			
Baseline	101.15 ± 3.90	101.00 ± 3.40	.732
4th week	101.55 ± 4.16	100.45 ± 3.03	.962
8th week	102.33 ± 3.96	100.03 ± 3.16	.264
12th week	102.50 ± 4.05	99.80 ± 3.28	.145
Waist to hip ratio (%)			
Baseline	0.83 ± 0.02	0.84 ± 0.03	.676
4th week	0.84 ± 0.03	0.83 ± 0.02	.476
8th week	0.84 ± 0.03	0.83 ± 0.02	.096
12th week	0.84 ± 0.03	0.83 ± 0.02	.052
Body fat (%)			
Baseline	35.51 ± 4.26	35.78 ± 4.23	.600
4th week	37.52 ± 5.04	34.88 ± 3.64	.166
8th week	38.69 ± 3.62	34.29 ± 3.52	.002*
12th week	38.73 ± 3.60	33.33 ± 4.35	<.001*
Lean body mass (%)			
Baseline	64.49 ± 4.26	64.23 ± 4.23	.601
4th week	62.52 ± 5.06	65.13 ± 3.64	.172
8th week	61.31 ± 3.62	65.72 ± 3.52	.002*
12th week	61.27 ± 3.63	66.66 ± 4.34	<.001*

Data are presented as mean ± SD. Work, occupation, and stress were adjusted. Bonferroni correction with adjusted *α*-level (*α*′ = 0.05/3 = 0.0167) was used for multiple comparisons between each time point and the baseline. No difference between a time point and the baseline was found.

*Significantly different between two groups, *P* < .05.

**Table 3:** Heart rate variability of the two study groups by mixed model.

Variables	Control (*n* = 20)	Experimental (*n* = 20)	*P-*value
HR (bpm)			
Baseline	76.15 ± 11.71	75.95 ± 9.46	.093
4th week	75.20 ± 11.46^†^	71.70 ± 10.00^†^	.399
8th week	78.65 ± 12.17^†^	71.30 ± 7.54^†^	.060
12th week	75.75 ± 7.95^†^	71.20 ± 7.14^†^	.331
SDNN (ms)			
Baseline	27.74 ± 12.63	28.95 ± 11.04	.609
4th week	25.29 ± 12.32	42.51 ± 21.62^†^	.024*
8th week	25.40 ± 13.25	37.47 ± 12.90	.029*
12th week	24.93 ± 12.75	37.84 ± 13.11	.105
RMSSD (ms)			
Baseline	28.44 ± 16.40	22.94 ± 17.02	.079
4th week	32.83 ± 24.97	19.48 ± 11.81	.082
8th week	27.68 ± 15.69	20.40 ± 16.21	.125
12th week	26.16 ± 11.84	20.61 ± 13.33	.629
Total power (ms^2^)			
Baseline	591.67 ± 479.69	830.37 ± 773.41	.225
4th week	589.58 ± 766.77	1837.12 ± 2060.79	.049*
8th week	608.76 ± 761.64	1160.05 ± 866.09	.066
12th week	553.31 ± 610.40	1272.76 ± 1126.29	.158
LF (ms^2^)			
Baseline	177.61 ± 205.10	244.26 ± 270.26	.409
4th week	186.34 ± 434.22	498.67 ± 522.62	.130
8th week	157.74 ± 244.64	346.60 ± 336.71	.114
12th week	162.73 ± 233.30	433.18 ± 533.66	.360
HF (ms^2^)			
Baseline	161.50 ± 229.62	185.87 ± 265.58	.643
4th week	113.33 ± 159.14	266.32 ± 383.74	.152
8th week	150.99 ± 232.14	183.19 ± 161.47	.420
12th week	127.57 ± 157.84	161.13 ± 120.27	.980
LF nu			
Baseline	52.76 ± 25.30	57.22 ± 20.96	.285
4th week	64.04 ± 16.35	54.35 ± 16.58	.441
8th week	61.84 ± 18.12	55.28 ± 16.56	.902
12th week	61.94 ± 18.93	54.94 ± 16.24	.725
HF nu			
Baseline	47.24 ± 25.30	42.78 ± 20.96	.285
4th week	35.96 ± 16.35	45.65 ± 16.58	.441
8th week	38.16 ± 18.12	44.72 ± 19.75	.902
12th week	38.06 ± 19.83	45.06 ± 16.24	.725
LF/HF ratio			
Baseline	2.24 ± 2.36	2.47 ± 3.31	.541
4th week	1.56 ± 1.18	3.06 ± 3.70	.299
8th week	1.99 ± 2.37	2.62 ± 2.94	.644
12th week	1.50 ± 0.87	2.60 ± 2.37	.345

HR, heart rate; SDNN, standard deviation of normal-to-normal RR intervals; RMSSD, the square root of the mean squared differences of successive NN intervals; LF, low frequency; HF, high frequency; LF nu, LF in normalized unit; HF nu, HF in normalized unit; Data were presented as mean ± SD. Work, occupation and working stress were adjusted. Bonferroni correction with adjusted *α*-level (*α*′ = 0.05/3 = 0.0167) was used for multiple comparisons between each time point and the baseline.

*Significantly different between two groups, *P* < .05; ^†^Significantly different between the present time point and the baseline, *P* < .0167.
